# Functional Nanocomposite Films of Poly(Lactic Acid) with Well-Dispersed Chitin Nanocrystals Achieved Using a Dispersing Agent and Liquid-Assisted Extrusion Process

**DOI:** 10.3390/molecules26154557

**Published:** 2021-07-28

**Authors:** Mitul Patel, Daniel Schwendemann, Giorgia Spigno, Shiyu Geng, Linn Berglund, Kristiina Oksman

**Affiliations:** 1 Division of Materials Science, Luleå University of Technology, SE-97 187 Luleå, Sweden; mitul.kumar.patel@ltu.se (M.P.); shiyu.geng@ltu.se (S.G.); linn.berglund@ltu.se (L.B.); 2 Institute for Material Engineering and Plastics Processing, University of Applied Sciences Eastern Switzerland, CH-8640 Rapperswil, Switzerland; daniel.schwendemann@ost.ch; 3 Department for Sustainable Food Process (DiSTAS), Università Cattolica del Sacro Cuore, Via Emilia Parmense 84, 29122 Piacenza, Italy; giorgia.spigno@unicatt.it; 4 Mechanical & Industrial Engineering, University of Toronto, Toronto, ON M5S 3BS, Canada

**Keywords:** chitin nanocrystals, nanocomposites, liquid-assisted extrusion, dispersion, melt strength, mechanical properties, antibacterial property, migration

## Abstract

The development of bio-based nanocomposites is of high scientific and industrial interest, since they offer excellent advantages in creating functional materials. However, dispersion and distribution of the nanomaterials inside the polymer matrix is a key challenge to achieve high-performance functional nanocomposites. In this context, for better dispersion, biobased triethyl citrate (TEC) as a dispersing agent in a liquid-assisted extrusion process was used to prepare the nanocomposites of poly (lactic acid) (PLA) and chitin nanocrystals (ChNCs). The aim was to identify the effect of the TEC content on the dispersion of ChNCs in the PLA matrix and the manufacturing of a functional nanocomposite. The nanocomposite film’s optical properties; microstructure; migration of the additive and nanocomposites’ thermal, mechanical and rheological properties, all influenced by the ChNC dispersion, were studied. The microscopy study confirmed that the dispersion of the ChNCs was improved with the increasing TEC content, and the best dispersion was found in the nanocomposite prepared with 15 wt% TEC. Additionally, the nanocomposite with the highest TEC content (15 wt%) resembled the mechanical properties of commonly used polymers like polyethylene and polypropylene. The addition of ChNCs in PLA-TEC15 enhanced the melt viscosity, as well as melt strength, of the polymer and demonstrated antibacterial activity.

## 1. Introduction

PLA is a member of the aliphatic polyester family produced from renewable resources and has recently gained attention due to its biocompatibility, biodegradability, transparency, high modulus and strength. In contrast to the above benefits, PLA is limited by its inherent brittleness, low melt strength, relatively low crystallization rate and moderate barrier properties, which hindered it from wide-spread applications, i.e., food packaging [1,2,3]. To overcome the above problems, nanocomposites reinforced with bio-based nanomaterials have recently attracted great attention in the last two decades due to environmental reasons, as well as the unique properties and functionalities of nanomaterials. The manufacturing processes of bionanomaterial-reinforced composites have been mainly carried out using solvent casting [4,5,6] and melt extrusion [7,8]. Due to its industrial adaptability, the later strategy for nanocomposite processing is of growing interest. However, the conventional extrusion process for the preparation of PLA nanocomposites is challenging due to the feeding of dried nano-sized materials. Bionanomaterials have a high tendency to aggregate and form micrometer-scale agglomerates during the drying process, which makes them difficult to redisperse. To improve the dispersion of nano-sized reinforcements in PLA polymer matrices, different approaches have been attempted, including surface modifications by acylation and polymer grafting, as well as the coupling agent, which enhance the compatibility between the nanomaterial and polymers [9,10,11]. These strategies, however, are expensive, complex, time-consuming and do not solve all of the problems described above. To overcome the above limitations, without the aid of any costly and time-consuming processes, we have successfully demonstrated in our previous studies [7,8,12,13,14] that the extrusion process using the liquid-assisted feeding of nanomaterials with processing aid (dispersing agent and solvent) is one way of avoiding the drying process and improving compatibility between the polymer and nanomaterial and, hence, improve their dispersion. Several researchers have reported the use of plasticizers as a dispersing agent of nanomaterials in the PLA matrix. For instance, Oksman et al. [13] showed a successful dispersion of cellulose nanocrystals in PLA when poly(ethylene glycol) (PEG) was used as a processing aid. Herrera et al. [14] demonstrated that cellulose nanofibers (CNFs) can be dispersed when the glycerol triacetate (GTA) plasticizer was used as a processing aid. Recently, Herrera et al. [15] used a biobased plasticizer, TEC (a citrate ester), for the dispersion of ChNCs in a PLA polymer and showed that increasing the TEC content up to 7.5 wt% had a positive effect. 

The use of a bio-based plasticizer such as TEC has received much attention recently owing to its nontoxic, biodegradable nature and also due to its good miscibility with PLA [15,16] because of the polar interactions between ester groups [17]. Apart from its dispersing aid ability, TEC is commercially used to improve the flexibility and toughness, as well as processability of PLA, to make it suitable for packaging and other applications. Several studies have reported that a higher amount of TEC (10–20 wt%) is required to obtain a noticeable plasticizing effect (flexibility and toughness) on PLA [8]. 

Chitin is found in crustacean shells; insect cuticles and the cell walls of yeast, fungi and green algae, where it is biocompatible, as well as biodegradable, with low cytotoxicity and antimicrobial activity [18]. Recently, chitin nanomaterials, chitin nanocrystals (ChNC) and chitin nanofibers (ChNFs) have received significant scientific interest due to the combination of mechanical and physicochemical properties. Besides, chitin nanomaterials possess a high aspect ratio (10–55), high surface area and reactive surface (–NHCOCH_3_ and –OH and residual –NH_2_ groups), which facilitate surface functionalization [19,20]. These beneficial properties have led to the utilization of chitin nanomaterials as reinforcements in PLA nanocomposites [7,21,22]. ChNCs are colloidal and rod-shaped, which represents the crystalline domain and is isolated from extracted chitin under certain specific conditions such as acidolysis [20]. Depending on the chitin source, ChNCs usually have a width ranging between 5 and 70 nm and a length between 150 and 800 nm. The Young’s modulus of ChNCs from α-chitin and β-chitin are ~41 GPa [23] and ~150 GPa [24], respectively, which indicate that they can provide a strong reinforcing effect on polymers. Several studies [21,25,26] have successfully demonstrated that bionanocomposites with a low loading of ChNCs can lead to materials with specific properties and high performances. Herrera et al. [21] found that the addition of only 1 wt% ChNCs in PLA imparts antifungal activity to the nanocomposite apart from its reinforcing advantage. Zhang et al. [25] reported an improvement in the mechanical properties of the PLA-ChNC nanocomposite by the addition of acetylated ChNCs. The functionalization of ChNCs with L-lactide presented by Li et al. [26] resulted in excellent tensile properties of the PLA-ChNC nanocomposite.

TEC can play an important role in the preparation of nanocomposites, where improved properties are enabled by the dispersion and distribution of the ChNCs. With respect to dispersion, the use of TEC as a dispersing aid has not been systematically evaluated to date, and only one study made in our research group has previously been published on this topic [7]. We demonstrated that the dispersion of ChNCs (3 wt%) was improved with the increasing TEC content (2.5, 5, 7.5 wt%), without plasticizing the PLA matrix. However, the conclusion was that a higher amount (≥7.5 wt%) of TEC is required to disperse the ChNCs in the PLA matrix, as well as improve the toughness of the material, thereby pinpointing the need for further research in this area. From all of the above literature reports, it is evident that the role of TEC in improving the specific properties of PLA, as well as the dispersion of nanomaterials in PLA nanocomposites, is an interesting topic of the current research. 

The present study is aiming to identify the more optimal TEC content for improved dispersion of the ChNCs in the PLA matrix and manufacture functional nanocomposites. In the present study, the concentration of the TEC was varied from 7.5 to 15 wt% with a 2.5 wt% increment in combination with the ChNC content of 1 wt%. The liquid-assisted twin-screw extrusion was used to produce nanocomposite pellets, which were then compression-molded to films. The dispersion state of ChNCs in nanocomposites was analyzed using optical and material morphology; plasticizer migration and thermal, mechanical and rheological properties. The melt strength and antibacterial properties of the best-dispersed ChNCs nanocomposites were also evaluated in this study.

## 2. Results

### 2.1. ChNC Characteristics

**Figure 1:**
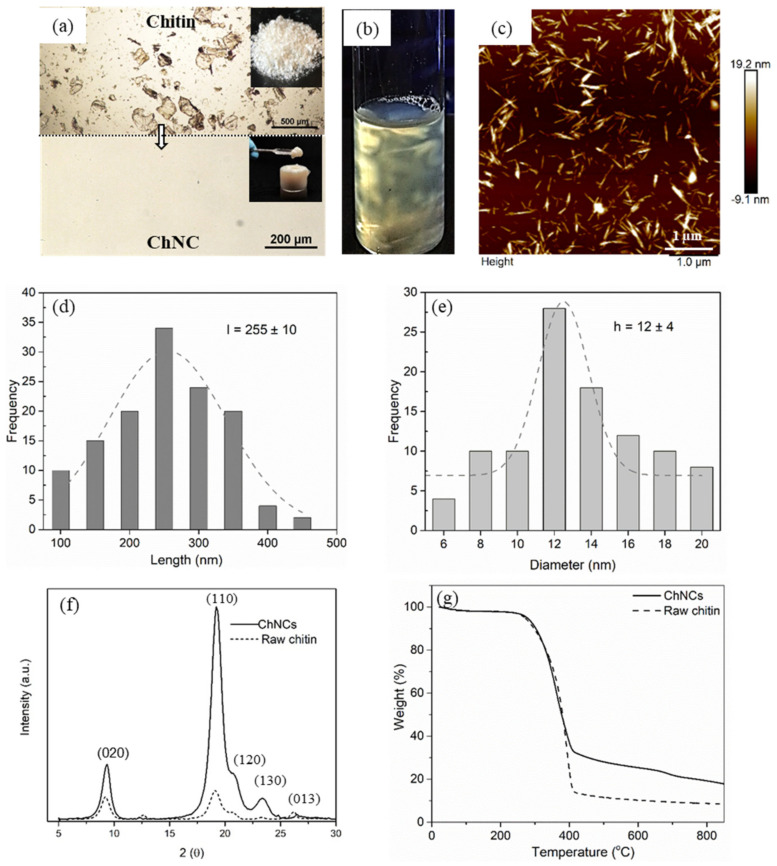
ChNC characterizations: (**a**) optical microscopy of chitin, ChNCs dispersed in water and a photograph of chitin powder and 18 wt% ChNC gel. (**b**) Flow birefringence pattern of ChNC suspension. (**c**) AFM height image of the ChNCs. (**d**) Length distributions of the ChNCs. (**e**) Diameter (height) distribution of the ChNCs. (**f**) XRD spectra of the raw chitin and ChNCs. (**g**) Thermogravimetric analysis of the raw chitin and ChNCs.

An optical microscopy (OM) image of chitin and isolated ChNCs dispersed in water is shown in Figure 1a and Figure S1. The OM images before and after the hydrolysis step indicate that the large chitin particle size was reduced into smaller particles, which are not visible in OM due to the resolution limit; however, this is an indication of a successful hydrolytic process. The inset of Figure 1a displays the appearance of this suspension in concentrated form (18 wt%). Figure 1b displays a flow birefringence pattern that shows the ability of nanocrystals to form a chiral nematic liquid crystalline phase in equilibrium with the isotropic phase [27] due to the presence of dispersed ChNCs; this is further confirming that successful hydrolysis was reached. 

The size and shape of ChNCs plays an important role in nanocomposite properties. Hence, AFM was performed to study the morphology of ChNCs, and the AFM micrograph in Figure 1c displays rod-shaped ChNCs. The size distributions of the obtained ChNCs are shown in Figure 1d,e. The length of them is in the range of 100–500 nm (average 255 nm), and the width is between 6 and 20 nm (average 12 nm). 

The XRD graph in Figure 1f of raw chitin and ChNC shows a strong diffraction peak at 2θ values of 9.2 (020) and 19.2 (110) and three weak peaks at 20.7 (021), 23.2 (130) and 26.2 (013), which correspond to the characteristic diffraction patterns of the α-chitin structure [28]. Compared to its native chitin, ChNCs showed diffraction peaks with higher intensities. The crystallinity index (CI) calculated using the XRD graph indicates that the produced ChNCs have higher crystallinity (93%) than native chitin (64%). The TGA thermograms of the chitin and ChNCs are shown in Figure 1g. Both raw chitin and ChNCs had a similar degradation onset at approximately 255 °C, and their main thermal degradation was observed around 300–400 °C. This confirms that the produced ChNCs were thermally stable at the extrusion processing temperature below 300 °C. The higher residual mass of the ChNCs at 900 °C compared to that of the raw chitin is likely due to their higher crystallinity. The crystalline phase of chitin is more closely packed than the amorphous phase, which may result in more crosslinking reactions at high temperatures, generating higher char contents and less volatiles; similar results were reported by Larbi et al. [29]. 

### 2.2. Determination of Dispersing Agent Amount

To determine the possible loss of TEC during the extrusion process, isothermal TGA thermograms were studied, as seen in Table 1 and Figure S1 (Supplementary Information). The mass loss below 8 min (~105 °C) is attributed to the removal of moisture, hence not considered. The TEC mass loss started after 15 min and was almost constant after 150 min for all the materials, indicating that the TEC evaporation process approaches completeness in the isothermal cycle (240 min). The total mass loss for all the samples at 180 °C is reported in Table 1. The difference in the TEC amount (added vs. removal amounts) indicates the loss of TEC during the melt extrusion process for all the materials. The difference is significant in materials with a higher amount of TEC. For instance, a material made with 15 wt% TEC shows 12.9 wt% and 11.8 wt% of TEC in the final composition of PLA-TEC15 and PLA-TEC15-ChNC, respectively.

It is noteworthy that the mass loss in the isothermal cycle was lower in the nanocomposites than in their counterparts. A possible reason could be that the influence of ChNCs on the plasticizer antimigration capability in the nanocomposite makes it difficult to degrade at an isothermal temperature.

### 2.3. Dispersion of ChNCs

**Figure 2:**
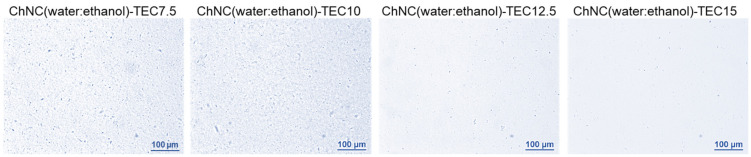
Optical microscopy of liquid feeding suspensions showing the dispersion of ChNCs in water, ethanol and varied concentrations of the dispersing agent.

To ensure that the feeding suspensions, prior to their use in the melt extrusion, are homogen and that the ChNCs are well-dispersed in the water:ethanol (1:5) TEC mixture, an optical microscopy study was performed. The micrographs of the suspensions are shown in Figure 2 and Figure S2 (Supplementary Information). Figure 2 shows that the water:ethanol ratio to TEC is appropriate, since there is no visible phase separation of the TEC in the mixture. Small agglomerates can be seen in the suspension with 7.5 and 10 wt% TEC, but these are not observed in the suspensions with the higher TEC contenta (12.5 and 15 wt%); this is also confirmed in Figure S2, Image analysis of the aggregate size distribution. The micrographs thus indicate that the feeding suspensions, particularly those with the higher TEC contents, have well-dispersed ChNCs, which is very important for the final result.

The optical transparency of the nanocomposite films was investigated to study the dispersion of ChNCs in the PLA matrix with the presence of TEC. It is known that the presence of agglomerates will scatter or disperse light in a nanocomposite film, contributing to decreased light transmittance [30]. Among the four nanocomposites, the highest transparency was seen in PLA-TEC15-ChNC and the lowest in PLA-TEC7.5-ChNC. It was noticed that the addition of ChNCs reduces the transmittance of the corresponding PLA-TEC films by 10.4% in PLA-TEC7.5-ChNC (from 91.5 to 82.0), 6.4% in PLA-TEC10-ChNC (from 91.6 to 85.7), 2.3% in PLA-TEC12.5-ChNC (from 90.8 to 88.7) and 2.1% in PLA-TEC15-ChNC (from 90.4 to 88.5); the results are summarized in Table S1 (Supplementary Information). The results show that the addition of 1 wt% ChNCs slightly decreased the transparency of the PLA-TEC films; however, the increasing content of the dispersing agent improved the transmittance of the nanocomposites from 82% to 88.5%, indicating a better dispersion of ChNCs in the nanocomposite. 

**Figure 3:**
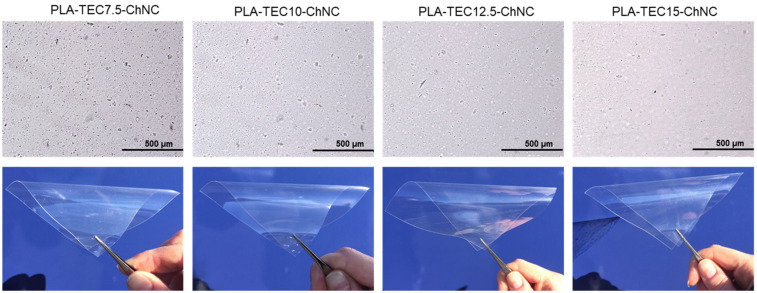
Optical microscopy images showing the dispersion of the ChNCs in nanocomposite films (on the **top**) and their corresponding photographs showing their visual appearance (on the **bottom**).

The optical microscopy and visual appearance of all the nanocomposite films are presented in Figure 3 and Figure S3 (Supplementary Information). The micrographs show some ChNC agglomerates in the nanocomposites with 7.5 and 10 wt% TEC, and the agglomerate size decreases so that fewer agglomerates are visible in the nanocomposites with 12.5 and 15 wt% TEC. This is also shown in Figure S3, Image analysis of the aggregate size distribution. The results confirm that an increased TEC content enhances the ChNC dispersion in the nanocomposites. However, the difference in transparency cannot be identified by visual appearance; the photographs in Figure 3 indicate that all the nanocomposites film appeared colorless and retained their transparency. 

**Figure 4:**
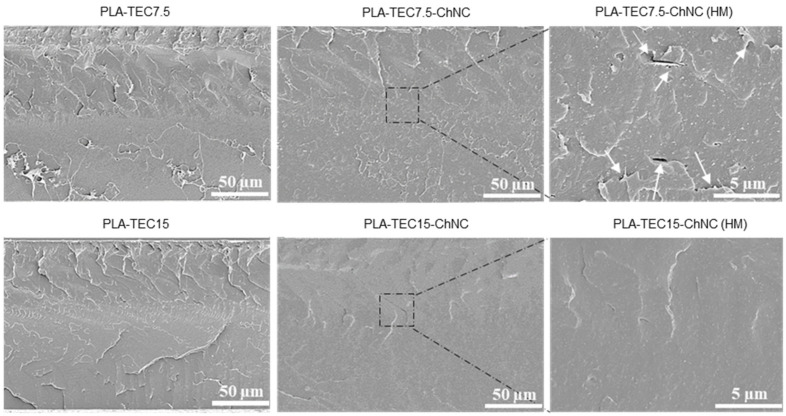
High-resolution SEM images of the cryofractured surface of the material prepared with 7.5 and 15 wt% TEC display that the pore formation due to the presence of agglomerates in PLA-TEC7.5-ChNC, while PLA-TEC15-ChNC possesses a smooth surface due to a better dispersion of ChNCs (HM: higher magnification).

To compare, the morphology of the cryogenic fractured surfaces of PLA-TEC7.5, PLA-TEC15 and their respective nanocomposites was investigated by high-resolution SEM, as shown in Figure 4. The micrographs at higher magnification revealed that there is a better dispersion of ChNCs in PLA-TEC15.0-ChNC, as shown in Figure 4. Additionally, white arrows in the high-magnification (HM) micrograph of PLA-TEC7.5-ChNC shows the presence of ChNC agglomerates, which have resulted in voids in the sample preparation. Moreover, in contrast to the rough surface of PLA-TEC7.5-ChNC, illustrated in Figure 4, the PLA-TEC15-ChNC nanocomposite exhibits a smoother and more homogenous surface without any evidence of ChNC aggregates as the sample with a lower TEC content. It seemed reasonable that the dispersing agent possibly covers the dispersed ChNCs in the liquid phase, thus preventing their agglomerates in the extrusion process; however, to cover all ChNC surfaces, a sufficient amount of dispersing agent is needed.

**Figure 5:**
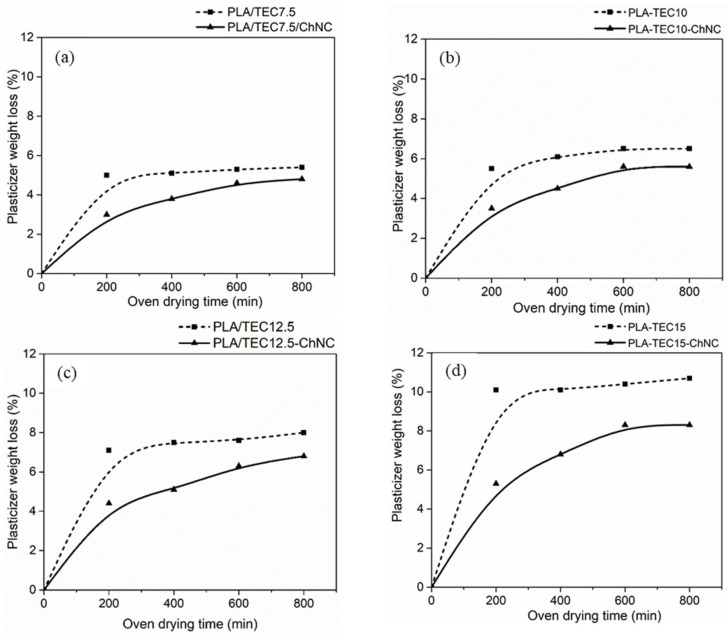
Migration test of the materials. (**a**) Polymer and its nanocomposite with 7.5 TEC, (**b**) with 10 TEC, (**c**) with 12.5 TEC and (**d**) with 15 TEC showing the effect of ChNC dispersion on the migration of TEC.

A migration analysis (Figure 5) was carried out to study the effect of dispersed ChNCs on the migration of TEC from the nanocomposites. Overall, TEC migration mostly happened in the initial stages (~200 min), and equilibrium conditions were reached after 400 min of the experiment. For the PLA-TEC materials, the weight loss was directly proportional to the TEC concentration in PLA. Whereas the addition of ChNCs significantly restricted the TEC to migrate, and the reduction effect was higher with the increasing amount of TEC from 0.86% to 2.36%. Similarly, Li et al. [31] reported a plasticizer migration reduction by adding nano CaCO_3_ and organic montmorillonite (OMMT) in polyvinyl chloride (PVC) due to the large specific surface area of the nanoparticles, which provided a strong adsorption force and migration inhibition. Ma et al. [32] also reported that the nanoparticles restrict the movement of plasticizer molecules by providing a higher steric resistance. The nanocomposites with dispersed ChNCs exhibit a higher ChNC-specific surface area than the nanocomposites with ChNC agglomerates. Therefore, the migration reduction was more significant in PLA-TEC15-ChNC because of the larger specific surface area generating from the better dispersion of ChNCs. Whereas, in the nanocomposites with a lower amount of TEC (7.5%, 10% and 12.5%), more interhydrogen bonds between crystals were predominant due to ChNC agglomerates, resulting in less interactions with TEC, which was responsible for more TEC migration.

**Figure 6:**
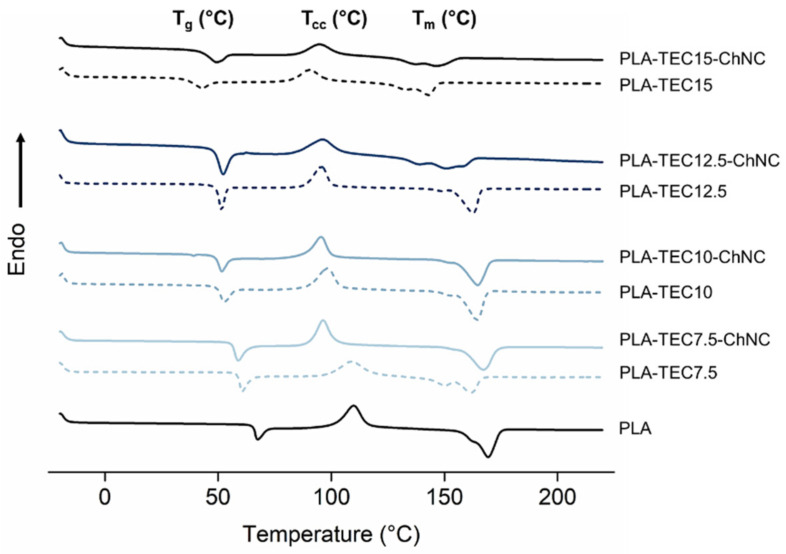
DSC thermograms of PLA-TEC films and their nanocomposites showing the effect of ChCNs on the thermal properties of the nanocomposites.

The thermal properties of the PLA, PLA-TEC and their respective nanocomposites were investigated and reported in Table 2, and the comparative thermograph is illustrated in Figure 6. Neat PLA showed a typical glass transition peak (T_g_), cold crystallization peak (T_cc_) and melting peak (T_m_) at 65 °C, 101 °C and 169 °C, respectively, which decreased subsequently with the addition of TEC due to the increased molecular chain mobility of the polymer. For example, the T_g_ was decreased from 65.3 (PLA) to 36 °C (PLA-TEC15), and the T_m_ was reduced from 169.4 (PLA) to 143 °C (PLA-TEC15) by the increasing amount of TEC.

For the nanocomposites, the value of T_g_ did not increase with the addition of ChNCs in PLA with TEC amounts from 7.5 to 12.5 wt% compared to their reference PLA compositions, whereas the T_g_ of PLA-TEC15-ChNC increased from 36 to 42 °C compared to PLA-TEC15. Similarly, the improvement in T_cc_ onset, T_m_ and crystallinity was seen in the nanocomposites with 15 wt% TEC. The significant improvement in the thermal properties of PLA-TEC15-ChNC is ascribed to the fact that well-dispersed ChNCs in PLA-TEC and good interfacial interactions between the ChNCs and PLA-TEC restrict polymer chains’ molecular mobility and, hence, result in a change of the T_g_ towards a higher temperature [7].

The mechanical properties, including Young’s modulus, tensile strength, elongation at break and work of the fracture of the prepared materials, are summarized in Table 3. The representative stress–strain curves for all materials and a comparative graph of the Young’s modulus and tensile strength are illustrated in Figure S4 (Supplementary Information). The results show that the addition of TEC reduces the tensile strength and Young’s modulus in all concentrations due to the plasticizing effect. Whereas the addition of TEC effectively enhanced the flexibility and ductility of the PLA, which led to tougher material. Up to a TEC content of 12.5 wt%, the changes in the Young’s modulus, tensile strength and elongation at the break of PLA were minor, but the highest TEC content (PLA-TEC15) significantly affected the mechanical properties of the PLA. The Young’s modulus and tensile strength reduced to 1.01 GPa and 27.6 MPa, respectively, whereas the elongation at the break increased to 311%, which was almost five-fold higher than the PLA-TEC12.5 and a 49-fold increment over the neat PLA. The elongation at the break of PLA-TEC15 was significantly higher compared to the previously reported (24.5%) value by Herrera et al. [14], where 20 wt% GTA was used as a processing aid for the dispersion of 1 wt% cellulose nanofiber in PLA.

Moreover, the addition of 1 wt% ChNCs in this PLA-TEC15 resulted in an increased Young’s modulus (from 1.01 to 1.41 GPa), along with a comparable elongation to break and work of the fracture compared with the reference material. These improvements may be attributed to better dispersion of the ChNCs and then contribute to better mechanical properties. 

PLA-TEC15 and its nanocomposite PLA-TEC15-ChNC resemble the properties of polymers like polypropylene (PP) and low-density polyethylene (LDPE), which are commonly used in packaging applications, as shown in Table 4. 

However, the addition of low molecular weight TEC causes an increase in the free volume and in chain mobility of the PLA, resulting in a decreased T_g_. As a result, the melt viscosity and melt strength (viscoelasticity) of PLA-TEC15 decrease with the increased melt flow. The melt strength is one of the most important factors while evaluating the processability of polymers, especially in the film-blowing process [34]. Interestingly, the addition of 1 wt% ChNC enhances the melt strength of PLA-TEC15, shown in Supplementary Video S1. From the video, it is obvious that the melt of PLA-TEC15 pressed out from the capillary rheometer deformed and broke because of its own weight, whereas the addition of 1 wt% ChNCs kept it intact without breakage because of the improved melt strength of PLA-TEC15-ChNC. This behavior indicates that well-dispersed ChNCs form an interconnected network-like structure in PLA that increases its resistance to deformation. A similar behavior was reported by Shojaeiarani et al. [35], in which there was a significant improvement in the melt strength of the PLA matrix by the addition of surface-modified cellulose.

**Figure 7:**
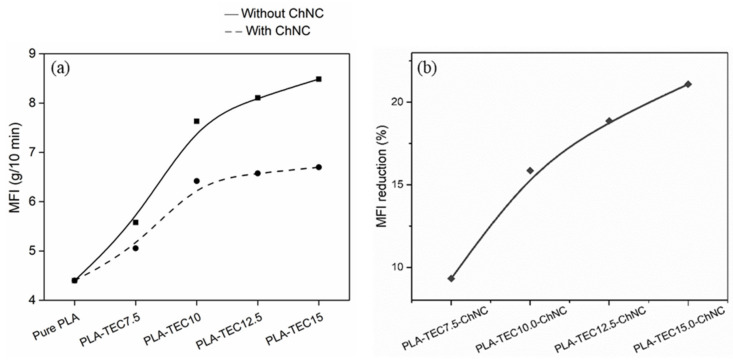
(**a**) Effect of ChNC on the melt flow index. (**b**) MFI reduction in nanocomposites in reference to their counterpart PLA-TEC.

MFI of the produced materials is indirect information of the interaction between the polymer, dispersion agent and ChNCs, as the melt polymer flow is affected by the interfacial characteristics due to the change in viscosity. Herrera et al. [7] reported that the addition of TEC improved the intramolecular interaction between PLA and ChNCs. The test was performed to study the effect of the addition of ChNCs in combination with varying amounts of TEC on the flow properties of the polymer. The MFI values are summarized in Table S2 (Supplementary Information). The MFI value is higher in PLA with TEC than neat PLA due to the high polymer chain mobility, which decreased the polymer viscosity and allows them to flow easily. The material with the highest amount of TEC (PLA-TEC15) showed the highest MFI, as expected. The addition of the ChNCs decreased the MFI, as shown in Figure 7a. The reason can be that the strong internal bond of ChNCs might restrict the polymer chain mobility, thus increasing the polymer viscosity, which turned into a reduction in MFI. It is also noticeable in Figure 7b that the rate of decrease in MFI in nanocomposites slightly increased with the increasing amount of TEC. For instance, nanocomposites with 7.5, 10, 12.5 and 15 wt% TEC decreased the MFI by 9, 15, 19 and 21 wt%, respectively, in comparison to their respective counterpart dispersing agents. It indicates that the higher reduction in MFI could be due to better-dispersed ChNCs in PLA-TEC15-ChNC.

According to the ISO 22196:2011 standard, the term “antibacterial” describes the effect of an agent that suppresses the growth of bacteria on the surfaces of products. The antibacterial activity (R) is measured by using the difference in the logarithm of the viable cell count founded on an antibacterial-treated product and an untreated product. The antibacterial activity values can also be used to express the antibacterial activity as a % inhibition in the growth of the microorganism, comparing the treated specimen with the untreated one. 

Table 5 shows that the number of viable bacteria (CFU/mL) decreased (22% and 26% growth reduction in *S. aureus* and *E. coli*, respectively) in PLA-TEC15-ChNC due to the bactericidal effect of a small amount of ChNC. The antibacterial activity of ChNC could be due to positively charged ChNCs, which interact with negatively charged bacterial cell membranes, causing a leakage of protein and other intracellular constituents from bacteria [36]. The results showed a weak antibacterial activity compared to other materials tested in the literature. For example, Cottaz et al. [37] found R values of 1, 4 and 6 for PP, EVA and LLDPE films, respectively, incorporating isobutyl-4-hydroxybenzoate 2% wt/wt. However, increasing the ChNC percentage could improve the antibacterial activity.

## 3. Materials and Methods

### 3.1. Materials

Pelletized PLA (NatureWorks, grade Ingeo 4043 D, Mw: 199,000 g/mol [7]) purchased from Resinex Switzerland AG (Freienbach, Switzerland) was used as a matrix polymer for the nanocomposites, and TEC (98%) in liquid form (Mw: 276.3 g/mol) was purchased from VWR International AB (Spånga, Sweden) and used as a dispersion aid. Chitin powder from shrimp shells, grade C7170 was purchased from Sigma-Aldrich Sweden AB (Stockholm, Sweden), and hydrochloric acid (HCl, 37%) was supplied by Solveco AB (Rosersberg, Sweden) and used for the isolation of the ChNCs. Ethanol (99.5%) was supplied by Solveco (Stockholm, Sweden). 

### 3.2. Preparation of ChNCs and Suspensions for Liquid Feeding Process

The isolation process of the ChNCs was made according to a previous study, described by Salaberria et al. [38], with some modifications. In short, the hydrolysis was performed in 3-M HCl at 90 ± 5 °C, using continuous stirring for 90 min. The HCl-to-chitin ratio was 30 mL-to-1 g. After 90 min, the suspension was diluted with distilled water, followed by centrifugation (Beckman Coulter AB, Stockholm, Sweden) at 6000× *g* for 10 min and decanting the supernatant; the procedure was repeated until the suspension transformed into a colloidal state. Subsequently, the suspension was transferred to dialyze for 3 days until the pH of the suspension was stable. The last step was ultrasonication for 20 min (UP400S, Hielscher, Teltow, Germany) to disintegrate the remaining particles that were not isolated as a nanosize. The final ChNC-suspension was then evaporated to decrease its water concentration to a 18 wt% solid content; in this concentration, the ChNC suspension formed a gel; this gel was stored at 4 °C for later use.

**Figure 8:**
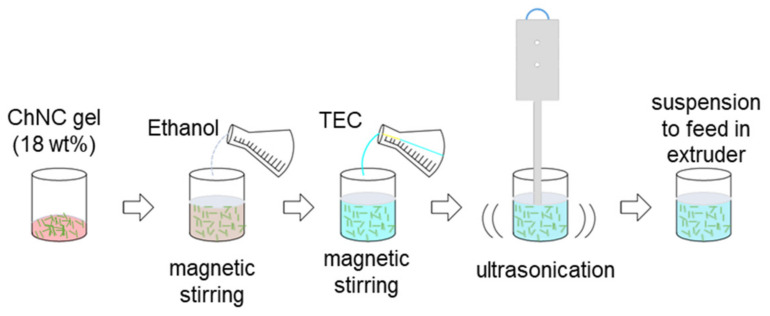
Schematic of the ChNC suspension preparation for liquid feeding.

The feeding suspension of the ChNCs was prepared as shown in Figure 8. The ChNC gel was first predispersed in ethanol using magnetic stirring for 2 h. The ethanol amount was taken according to the water content present in ChNC gel to maintain the water:ethanol ratio of 1:5. To make the final composition, the required amount of TEC was mixed into the suspension under the vigorous stirring conditions for 2 h. The suspension was ultrasonicated for 2 min before its use. The final quantity for producing the feeding suspension was dependent on the ChNC concentration. The higher concentration of ChNCs meant less liquid to be removed in the subsequent extrusion process. Hence, it is very important to increase the solid content as much as possible of the ChNC gel.

### 3.3. Liquid-Assisted Extrusion

**Figure 9:**
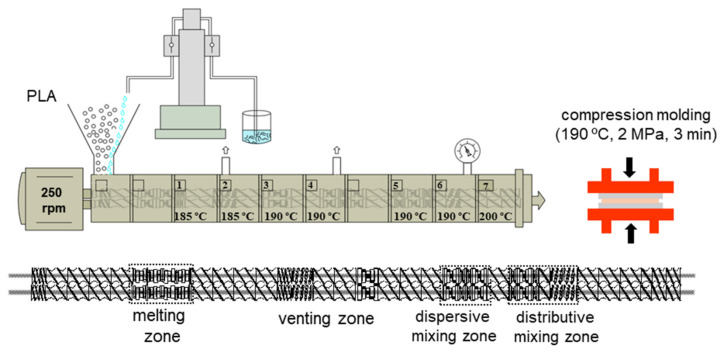
Schematic diagram of the liquid-assisted extrusion process displaying the process parameters and the twin-screw design showing different zones.

A corotating twin-screw extruder, with a L/D ratio of 40 and screw diameter of 18 mm (Coperion W&P ZSK-18 MEGALab, Stuttgart, Germany), was used for the compounding. The feeding of the PLA was made using a K-tron gravimetric feeder (Niederlenz, Switzerland), and a high-pressure syringe pump (500D, Teledyne Isco, Lincoln, NE, USA) was used for the feeding of the ChNC suspension. A schematic presentation of the process is shown in Figure 9. 

PLA granules and the liquid suspensions were fed at the main feeding zone with specific feeding rates, depending on the TEC concentration. The materials are coded as PLA-TEC*X* (*X* = 7.5, 10, 12.5 and 15), where the number indicates the dispersing agent concentration, and the ChNC content is kept constant at 1 wt%; the coding is shown in Table 6. The total throughput was 2 kg/h, the screw speed was 285 rpm and the range of the temperature profile was from 185 °C to 200 °C. The nancomposites were produced using the standard screw configuration, which includes melting, mixing and dispersing processing sections (see Figure 9). The extruder is equipped with two atmospheric venting in zones 2 and 4 and a vacuum venting unit zone 6; these eliminate the vapor generated in the processing of the liquid phases (water and ethanol 0.55 kg/h). The materials were extruded using a strand die; these strands were cooled in a water bath and then pelletized in a strand pelletizer and, finally, dried at 55 °C overnight. 

The extruded pellets were compression-molded using a LPC-300 Fontijne Grotnes press (Vlaardingen, Netherlands). Approximately, 4 g of the pellets were placed between aluminum plates covered with polymeric sheets (Mylar) and compression-molded at 190 °C initially for 2 min using a contact pressure and, then, with 2 MPa for another minute. Finally, the films were cooled immediately to room temperature to avoid crystallization. The obtained nanocomposite films were about 150 µm in thickness, which was then kept under ambient conditions for 24 h before characterizations.

### 3.4. Characterizations

#### 3.4.1. Characterizations of ChNCs

To validate the isolation process of the ChNCs from chitin, a Nikon Eclipse LV100N POL polarized optical microscope (Kanagawa, Japan) was used. Micrographs of the diluted suspension (0.1 wt%) before and after the isolation process were taken. An in-house flow birefringence setup was used to determine the presence and dispersion of the nanocrystals. The setup consists of a white light source and two polarized filters in a cross-direction. Diluted nanocrystals (0.5 wt%) under stirring condition were placed inside the chamber, and the image was taken through the cross-polarized filters.

The morphology of the ChNCs was studied by an atomic force microscope (AFM) Veeco Multimode Nanoscope V (Santa Barbara, CA, USA). AFM was performed at the resonance frequency of the antimony-doped silicon cantilevers (NCHV-A, Bruker, Camarillo, CA, USA) with a tip radius of 8 nm. All scans were performed in the air and tapping mode. The analysis of the average length and width of the ChNCs from the AFM micrographs was carried out using Gwyddion software [39].

The degree of crystallinity of raw chitin and isolated ChNCs was measured using an X-ray diffractometer (PAN analytical, Almelo, Netherlands) installed with a PIXcel^3d^ detector using Cu Ka radiation (λ = 0.154 nm). The following Segal Equation (1) (peak height method) was used to estimate the crystallinity index (CI) after baseline correction [40]:

(1) $$CI=\frac{I_{max}-I_{am}}{I_{max}}\times 100$$

where *I_max_* is the peak intensity at 2θ − 19.5°. *I_am_* is the height of the amorphous diffraction peak. To perform the extrusion process for polymer nanocomposites at high temperatures, the thermal stability of the chitin nanomaterial is important to be able to avoid thermal degradation. The thermal degradation behavior of the ChNCs was determined using a TA instrument, TGA-Q500 (TA Instruments, New Castle, DE, USA) in N_2_ atmosphere. The tests were performed in ramp mode from 25 to 900 °C at 10 °C/min.

#### 3.4.2. Characterization of Feeding Suspension and Nanocomposites

The presence of TEC content after the extrusion process was analyzed using TGA-Q500 (TA Instruments, New Castle, DE, USA). The temperature sweep was scheduled from 30 °C to 600 °C at a heating rate of 10 °C min^−1^ under a nitrogen atmosphere (flow rate of 60 mL/min). During this heating cycle, TGA in isothermal mode was performed at 180 °C for 240 min for the complete removal of TEC.

Analyses of the dispersion and distribution of ChNCs in liquid feeding suspension and nanocomposite films were carried out using a Nikon Eclipse LV100N POL polarized optical microscope (Kanagawa, Japan) and ImageJ analysis software (open source). Further, the fractured surface of nanocomposites films was studied using a high-resolution scanning electron microscope (SEM) FEI Magellan 400 XHR-SEM (FEI Company, Hillsboro, OR, USA) at an acceleration voltage of 5 kV and a current of 6.3 pA. The fracture cross-sections were sputter-coated (~10 nm) with platinum using EM ACE200, a Leica vacuum coater (Wetzlar, Germany), to avoid charging.

The light transmittance of the prepared films was studied using GENESYS, a 10-UV-Vis spectrophotometer (ThermoScientific, Bremen, Germany), at a visible wavelength of 550 nm. The film thickness was around 150 µm; the measurements were performed three times and reported as an average value.

The migration test was performed to understand the effect of ChNCs on the migration of TEC from the materials. Rectangular samples of 10 mm × 10 mm were kept at an isothermal temperature (100 °C) in a vented oven. These samples were taken out at regular time intervals of 200 min and wiped to clean the surface to remove the migrated dispersing agent, which was then weighed. The weight loss as a function of time at 100 °C was estimated as an average of three measured values [41]. The following Equation (2) is used to calculate the migration rate ($\omega_{t})$:

(2) $$\omega_{t}=\frac{m_{0}-m_{t}}{m_{0}}$$

where *m*_0_ and m_t_ are the masses of the sample before migration and migration after time *t*, respectively. 

Differential scanning calorimetry (DSC) was performed using a DSC 821e, Mettler Toledo (Schwerzenbach, Switzerland), with N_2_ as a carrier gas. The samples were performed in the temperature range of −20 to 220 °C, with a heating rate of 10 °C min^−1^. The crystallinity (*χ_c_*) of the samples was calculated following Equation (3):

(3) $$\chi_{c}\left(\%\right)=\frac{\Delta H_{m}-\Delta H_{cc}}{\Delta H_{m}^{ο}}\times \frac{100}{w}$$

where Δ*H_m_* is melting enthalpy, Δ*H_cc_* is cold crystallization enthalpy, $\Delta H_{m}^{ο}$ is the melting enthalpy of fully crystalline PLA (the value of 93 J/g was considered [42]) and w is the weight fraction of PLA.

The mechanical properties of the nanocomposites were characterized using a universal tensile testing machine, Shimadzu AG (Kyoto, Japan). Five rectangular specimens of 5.5 mm in width and 50 mm in length were cut from approx. 150-μm-thick films using a rectangular press mold. Samples were then conditioned for 24 h at 25 °C and a relative humidity (RH) of 50 before the testing. The used testing conditions were a 1-kN load cell, a gauge length of 20 mm and strain rate of 2 mm/min. The tensile strength and the elongation at break were obtained from the stress–strain data, and the E-modulus was calculated according to the slope of the initial linear part of the stress–strain curve.

A capillary rheometer (Rheo-tester 1000, Göttfert, Buchen, Germany) was used to study the melt strength of a polymer nanocomposite, which is defined as the drawdown force required to break a molten strand of the polymer. The molten polymer strand was pressed through the capillary rheometer equipped with a circular die with a 1-mm diameter 20 mm long and 0° entry angle at the temperature of 190 °C using the same piston velocity of 0.32 mm/s, and the film was recorded to compare the melt strength of PLA-TEC and their respective nanocomposite.

The material’s melt flow (MFI) properties were measured using MI1 Göttfert (Buchen, Germany). The pellets were isothermally extruded for 10 min under a constant load through a standard dimension die at 190 °C temperature and a load of 2.16 kg (ASTM D1238 standard and EN ISO 1133, method A). The process was repeated at least three times and the average value was reported in grams per 10 min. 

The antibacterial activity of the films (PLA, PLA-TEC15 and PLA-TEC15-ChNC) was assessed according to the method reported by the ISO 22196:2011 standard. Staphylococcus aureus (ATCC 8739) and Escherichia coli (ATCC 6538P) were used as the bacterial strains. The test was carried out with a bacterial suspension at a concentration of 6 × 10^5^ cells/mL CFU/mL. Test specimens were placed into a different sterile petri dish inoculated with the strains spread over the entire surface of the specimens, which were then kept for incubation at (35 ± 1) °C and 90% relative humidity for 1 day. The number of colony-forming units (CFU/mL) from the samples before and after incubation was determined by serial dilution and plate count method. The antibacterial activity (R) was calculated following Equation (4):

R = Ut − At(4)

where Ut is the average of the logarithm to the number of viable bacteria (CFU/mL) recovered from the untreated samples (PLA specimens) after 1 day, while At is the average of the logarithm to the number of viable bacteria (CFU/mL) recovered from the PLA-TEC15 and PLA-TEC15-ChNC specimens after 1 day.

## 4. Conclusions

The aim of this study was to evaluate the effect of different concentrations of triethyl citrate (TEC) as a dispersing agent of 1 wt% chitin nanocrystals (ChNCs) in the poly (lactic acid) PLA matrix. The materials were successfully prepared via a liquid-assisted extrusion process. 

The results confirmed that the dispersing agent plays an important role in improving the dispersion of the ChNCs in the PLA matrix. From the optical microscopy study, it was found that the dispersion of ChNCs was improved with increasing the TEC content, and a very small number of agglomerates were visible in the PLA-TEC15-ChNC film compared to the other nanocomposites. Moreover, the nanocomposite with 15 wt% TEC showed the highest improvement in the transparency value (from 82% to 88.5%) among all the nanocomposites, whereas no agglomerates were visible on the scanning electron micrographs, which indicated a better dispersion of the ChNCs. Nanocomposites with 15% TEC resemble the mechanical properties of commonly used petroleum-based polymers like PE and PP. Further, it was observed that dispersed ChNCs in PLA-TEC15 could act as multifunctional additives. For instance, it increases the melt viscosity, as well as enhancing the melt strength of the polymer, which are important parameters in polymer processing, especially in film blowing. Interestingly, due to the addition of 1 wt% ChNC, the PLA-TEC15-ChNC film exhibited an antibacterial effect on both S. aureus and E. coli, which is an important parameter in food packaging applications, improving food safety and prolonging shelf lives.

The study has shown that PLA nanocomposites can be successfully prepared using liquid-assisted extrusion, where a suspension of dispersing agent, water and ethanol and ChNCs are pumped into the polymer melt. Therefore, this process could be the next step for the production of nanocomposites on a large scale.

## Figures and Tables

**Table 1:** Mass loss of TEC from the nanocomposites at the isothermal temperature.

Materials	TEC Content (wt%)	
	Added	Mass Loss TGA
PLA-TEC7.5	7.5	7.1
PLA-TEC7.5-ChNC	7.5	6.8
PLA-TEC10	10	9.4
PLA-TEC10-ChNC	10	9.3
PLA-TEC12.5	12.5	11.0
PLA-TEC12.5-ChNC	12.5	9.9
PLA-TEC15	15	12.9
PLA-TEC15-ChNC	15	11.8

**Table 2:** Thermal properties of the polymer and its nanocomposites.

Materials	T_g_ (°C)	T_cc_ Onset (°C)	T_m_ (°C)	Crystallinity (%)
PLA	65.3	101.7	169.4	1.8
PLA-TEC7.5	58.0	98.9	161.7	2.4
PLA-TEC7.5-ChNC	55.9	90.6	167.4	2.6
PLA-TEC10	48.7	89.8	164.4	3.9
PLA-TEC10-ChNC	48.0	88.0	164.0	4.3
PLA-TEC12.5	47.1	88.9	153.0	6.0
PLA-TEC12.5-ChNC	47.1	84.0	154.0	6.4
PLA-TEC15	36.0	82.0	143.0	5.8
PLA-TEC15-ChNC	42.0	85.0	146.4	7.1

**Table 3:** Mechanical properties of PLA, PLA with TEC and the corresponding nanocomposites.

Materials	Young’s Modulus (GPa)	Tensile Strength (MPa)	Elongation at Break (%)	Work of Fracture (MJ/m^3^)
PLA	1.85 ± 0.03 ^a^	59.9 ± 0.5 ^a^	6.3 ± 0.7 ^a^	2.0 ± 0.4 ^a^
PLA-TEC7.5	1.76 ± 0.05 ^a,b^	46.0 ± 2.7 ^b^	10.2 ± 2.1 ^a,c^	3.8 ± 0.2 ^a,c^
PLA-TEC7.5-ChNC	1.82 ± 0.03 ^a,b^	49.0 ± 1.8 ^b^	8.7 ± 3.35 ^a^	2.3 ± 0.2 ^a^
PLA-TEC10	1.69 ± 0.02 ^c^	41.3 ± 1.5 ^c^	21.4 ± 2.8 ^a,c^	6.4 ± 0.9 ^b^
PLA-TEC10-ChNC	1.73 ± 0.02 ^b^	41.8 ± 1.2 ^c^	14.9 ± 2.3 ^a,c^	5.3 ± 1.4 ^b,c^
PLA-TEC12.5	1.61 ± 0.11 ^c^	39.4 ± 2.7 ^c^	65.7 ± 18.7 ^b^	16.1 ± 2.0 ^d^
PLA-TEC12.5-ChNC	1.62 ± 0.04 ^c^	40.9 ± 0.9 ^c^	24.1 ± 5.5 ^c^	6.6 ± 1.7 ^b^
PLA-TEC15	1.01 ± 0.08 ^d^	27.6 ± 3.8 ^d^	310.8 ± 56.4 ^α^	49.4 ± 6.3 ^α^
PLA-TEC15-ChNC	1.41 ± 0.10 ^e^	31.4 ± 0.9 ^d^	256.2 ± 15.5 ^α^	46.2 ± 2.4 ^α^

Note: The same superscript letters within the same column and material are not significantly different at the 5% significance level based on ANOVA and Tukey’s HSD multiple comparison test.

**Table 4:** Mechanical properties of commonly used petroleum-based polymers.

Materials	Young’s Modulus (GPa)	Tensile Strength (MPa)	Elongation at Break (%)
PLA-TEC15	1.01	27.6	310.8
PLA-TEC15-ChNC	1.41	31.4	256.2
LDPE [33]	0.17–0.28	8.3–31.4	100–650
PP [33]	1.14–1.55	31–41.4	100–600

**Table 5:** Antibacterial properties of PLA-TEC15-ChNC film showing an antibacterial activity against common microorganisms.

Material	*Staphylococcus aureus*		*Escherichia coli*	
	% Microbial Death	R	% Microbial Death	R
PLA-TEC15	-	-	17	0.08
PLA-TEC15-ChNC	22	0.11	26	0.13

**Table 6:** Parameters for the liquid-assisted extrusion process and composition of the targeted materials.

Coding	Composition of Materials (wt%)			Feeding Rate (kg/hr)	
	PLA	TEC	ChNC	PLA	Suspension
PLA	100	-	-	2.00	-
PLA-TEC7.5	92.5	7.5	-	1.85	0.70 ^1^
PLA-TEC7.5-ChNC	91.5	7.5	1	1.83	0.72 ^2^
PLA-TEC10	90.0	10	-	1.80	0.75 ^1^
PLA-TEC10-ChNC	89.0	10	1	1.78	0.77 ^2^
PLA-TEC12.5	87.5	12.5	-	1.75	0.80 ^1^
PLA-TEC12.5-ChNC	86.5	12.5	1	1.73	0.82 ^2^
PLA-TEC15	85.0	15	-	1.70	0.85 ^1^
PLA-TEC15-ChNC	84.5	15	1	1.68	0.87 ^2^

^1^ Suspension made up of water, ethanol and TEC. ^2^ Suspension made up of water, ethanol, TEC and ChNCs. The evaporation rate of the water and ethanol was held constant for each material, 0.55 kg/h.

## Data Availability

Available in request.

## Supplementary Materials

The following are available online. Figure S1. Isothermal TGA of the prepared materials. Figure S2. Image analysis of the ChNC dispersion in the feeding suspension. Figure S3. Image analysis of the CHNC dispersion in the nanocomposite films. Figure S4. Tensile properties of the plasticized materials and their respective counterpart nanocomposites. Table S1. Transmittance of neat PLA, plasticizes and nanocomposites. Table S2. Melt flow index of a neat PLA, plasticizes and nanocomposites. Video S1: Melt strength.

## Sample Availability

No avaiablity of samples.

## References

[1] Soulestin J., Prashantha K., Lacrampe M.F., Krawczak P. (2011). Bioplastics Based Nanocomposites for Packaging Applications. Handbook of Bioplastics and Biocomposites Engineering Applications.

[2] Kulinski Z., Piorkowska E. (2005). Crystallization, structure and properties of plasticized poly(L-lactide). Polymer.

[3] Lim L.T., Auras R., Rubino M. (2008). Processing technologies for poly(lactic acid). Prog. Polym. Sci..

[4] Salaberria A.M., Diaz R.H., Labidi J., Fernandes S.C.M. (2015). Preparing valuable renewable nanocomposite films based exclusively on oceanic biomass-Chitin nanofillers and chitosan. React. Funct. Polym..

[5] Gopalan Nair K., Dufresne A. (2003). Crab shell chitin whisker reinforced natural rubber nanocomposites. 1. Processing and swelling behavior. Biomacromolecules.

[6] Tran T.H., Nguyen H.L., Hwang D.S., Lee J.Y., Cha H.G., Koo J.M., Hwang S.Y., Park J., Oh D.X. (2019). Five different chitin nanomaterials from identical source with different advantageous functions and performances. Carbohydr. Polym..

[7] Herrera N., Singh A.A., Salaberria A.M., Labidi J., Mathew A.P., Oksman K. (2017). Triethyl citrate (TEC) as a dispersing aid in polylactic acid/chitin nanocomposites prepared via liquid-assisted extrusion. Polymers.

[8] Herrera N., Salaberria A.M., Mathew A.P., Oksman K. (2016). Plasticized polylactic acid nanocomposite films with cellulose and chitin nanocrystals prepared using extrusion and compression molding with two cooling rates: Effects on mechanical, thermal and optical properties. Compos. Part A Appl. Sci. Manuf..

[9] Arias A., Heuzey M.C., Huneault M.A., Ausias G., Bendahou A. (2015). Enhanced dispersion of cellulose nanocrystals in melt-processed polylactide-based nanocomposites. Cellulose.

[10] Salaberria A.M., Diaz R.H., Andrés M.A., Fernandes S.C.M., Labidi J. (2017). The antifungal activity of functionalized chitin nanocrystals in poly (Lactid Acid) films. Materials.

[11] Raquez J.M., Murena Y., Goffin A.L., Habibi Y., Ruelle B., DeBuyl F., Dubois P. (2012). Surface-modification of cellulose nanowhiskers and their use as nanoreinforcers into polylactide: A sustainably-integrated approach. Compos. Sci. Technol..

[12] Bondeson D., Oksman K. (2007). Polylactic acid/cellulose whisker nanocomposites modified by polyvinyl alcohol. Compos. Part A Appl. Sci. Manuf..

[13] Oksman K., Mathew A.P., Bondeson D., Kvien I. (2006). Manufacturing process of cellulose whiskers/polylactic acid nanocomposites. Compos. Sci. Technol..

[14] Herrera N., Mathew A.P., Oksman K. (2015). Plasticized polylactic acid/cellulose nanocomposites prepared using melt-extrusion and liquid feeding: Mechanical, thermal and optical properties. Compos. Sci. Technol..

[15] Labrecque L.V., Kumar R.A., Davé V., Gross R.A., Mccarthy S.P. (1997). Citrate esters as plasticizers for poly(lactic acid). J. Appl. Polym. Sci..

[16] Ljungberg N., Wesslén B. (2003). Tributyl citrate oligomers as plasticizers for poly (lactic acid): Thermo-mechanical film properties and aging. Polymer.

[17] Ren Z., Dong L., Yang Y. (2006). Dynamic mechanical and thermal properties of plasticized poly(lactic acid). J. Appl. Polym. Sci..

[18] Kurita K. (2006). Chitin and chitosan: Functional biopolymers from marine crustaceans. Mar. Biotechnol..

[19] Salaberria A.M., Labidi J., Fernandes S.C.M. (2015). Different routes to turn chitin into stunning nano-objects. Eur. Polym. J..

[20] Zeng J.B., He Y.S., Li S.L., Wang Y.Z. (2012). Chitin whiskers: An overview. Biomacromolecules.

[21] Herrera N., Roch H., Salaberria A.M., Pino-Orellana M.A., Labidi J., Fernandes S.C.M., Radic D., Leiva A., Oksman K. (2016). Functionalized blown films of plasticized polylactic acid/chitin nanocomposite: Preparation and characterization. Mater. Des..

[22] Li J., Gao Y., Zhao J., Sun J., Li D. (2017). Homogeneous dispersion of chitin nanofibers in polylactic acid with different pretreatment methods. Cellulose.

[23] Nishino T., Matsui R., Nakamae K. (1999). Elastic modulus of the crystalline regions of chitin and chitosan. J. Polym. Sci. Part B Polym. Phys..

[24] Paillet M., Dufresne A. (2001). Chitin whisker reinforced thermoplastic nanocomposites. Macromolecules.

[25] Zhang Q., Wei S., Huang J., Feng J., Chang P.R. (2014). Effect of surface acetylated-chitin nanocrystals on structure and mechanical properties of poly(lactic acid). J. Appl. Polym. Sci..

[26] Li C., Liu H., Luo B., Wen W., He L., Liu M., Zhou C. (2016). Nanocomposites of poly(l-lactide) and surface-modified chitin whiskers with improved mechanical properties and cytocompatibility. Eur. Polym. J..

[27] Fleming K., Gray D.G., Matthews S. (2001). Cellulose crystallites. Chem. Eur. J..

[28] Naseri N., Algan C., Jacobs V., John M., Oksman K., Mathew A.P. (2014). Electrospun chitosan-based nanocomposite mats reinforced with chitin nanocrystals for wound dressing. Carbohydr. Polym..

[29] Larbi F., García A., del Valle L.J., Hamou A., Puiggalí J., Belgacem N., Bras J. (2018). Comparison of nanocrystals and nanofibers produced from shrimp shell α-chitin: From energy production to material cytotoxicity and Pickering emulsion properties. Carbohydr. Polym..

[30] Oksman K., Mathew A.P., Oksman K., Mathew A.P., Bismarck A., Rojas O., Sain M. (2014). Melt Compounding Process of Cellulose Nanocomposites. Handbook of Green Materials: 2 Bionanocomposites: Processing, Characterization and Properties.

[31] Li X., Xiao Y., Wang B., Tang Y., Lu Y., Wang C. (2012). Effects of poly(1,2-propylene glycol adipate) and nano-CaCO_3_ on DOP migration and mechanical properties of flexible PVC. J. Appl. Polym. Sci..

[32] Ma Y., Liao S., Li Q., Guan Q., Jia P., Zhou Y. (2020). Physical and chemical modifications of poly(vinyl chloride) materials to prevent plasticizer migration-Still on the run. React. Funct. Polym..

[33] Callister W.D. (2015). Materials Science and Engineering: An Introduction.

[34] Liu X., Yu L., Dean K., Toikka G., Bateman S., Nguyen T., Yuan Q., Filippou C. (2013). Improving Melt Strength of Polylactic Acid. Int. Polym. Process.

[35] Shojaeiarani J., Bajwa D.S., Stark N.M., Bajwa S.G. (2019). Rheological properties of cellulose nanocrystals engineered polylactic acid nanocomposites. Compos. Part B Eng..

[36] Azeredo H.M.C. (2009). Nanocomposites for Food Packaging Applications. Food Res. Int..

[37] Cottaz A., Bouarab L., De Clercq J., Oulahal N., Degraeve P., Joly C. (2019). Potential of incorporation of antimicrobial plant phenolics into polyolefin-based food contact materials to produce active packaging by melt-blending: Proof of concept with isobutyl-4-hydroxybenzoate. Front. Chem..

[38] Salaberria A.M., Labidi J., Fernandes S.C.M. (2014). Chitin nanocrystals and nanofibers as nano-sized fillers into thermoplastic starch-based biocomposites processed by melt-mixing. Chem. Eng. J..

[39] Nečas D., Klapetek P. (2012). Gwyddion: An open-source software for SPM data analysis. Cent. Eur. J. Phys..

[40] Segal L., Creely J.J., Martin A.E., Conrad C.M. (1959). An Empirical Method for Estimating the Degree of Crystallinity of Native Cellulose Using the X-ray Diffractometer. Text. Res. J..

[41] Maiza M., Benaniba M.T., Quintard G., Massardier-Nageotte V. (2015). Biobased additive plasticizing Polylactic acid (PLA). Polimeros.

[42] Vasanthakumari R., Pennings A.J. (1983). Crystallization kinetics of poly(l-lactic acid). Polymer.

