# Supplementary material for: Functional Nanocomposite Films of Poly(Lactic Acid) with Well-Dispersed Chitin Nanocrystals Achieved Using a Dispersing Agent and Liquid-Assisted Extrusion Process
Source: Molecules. 2021 Jul 28;26(15):4557. doi: 10.3390/molecules26154557 (PMC8347658; doi:10.3390/molecules26154557)
Supplement: Supplementary file 1 [file molecules-26-04557-s001.zip › molecules-1256931-supplementary.pdf]

Supplementary information

Functional nanocomposite films of poly(lactic acid) with well-dispersed chitin nanocrystals achieved using a dispersing agent and liquid-assisted extrusion process

Mitul Patel, Daniel Schwendemann, Giorgia Spigno, Shiyu Geng, Linn Berglund and Kristiina Oksman

Table S1 Transmittance of neat PLA, compared with TEC plasticized PLA and nanocomposites.

Materials	Transmittance (%)
PLA	93.1 ± 0.1
PLA-TEC7.5	91.5 ± 0.4
PLA-TEC7.5-ChNC	82.0 ± 1.6
PLA-TEC10	91.6 ± 0.2
PLA-TEC10-ChNC	85.7 ± 0.2
PLA-TEC12.5	90.8 ± 0.3
PLA-TEC12.5-ChNC	88.7 ± 0.1
PLA-TEC15	90.4 ± 0.1
PLA-TEC15-ChNC	88.5 ± 0.1

Table S2 Melt flow index of neat PLA, TEC plasticized PLA and nanocomposites.

Materials	MFI (g/10 min)
PLA	4.4 ± 0.0
PLA-TEC7.5	5.6 ± 0.1
PLA-TEC7.5-ChNC	5.1 ± 0.1
PLA-TEC10	7.6 ± 0.0
PLA-TEC10-ChNC	6.4 ± 0.0
PLA-TEC12.5	8.1 ± 0.0
PLA-TEC12.5-ChNC	6.6 ± 0.0
PLA-TEC15	8.5 ± 0.0
PLA-TEC15-ChNC	6.7 ± 0.0

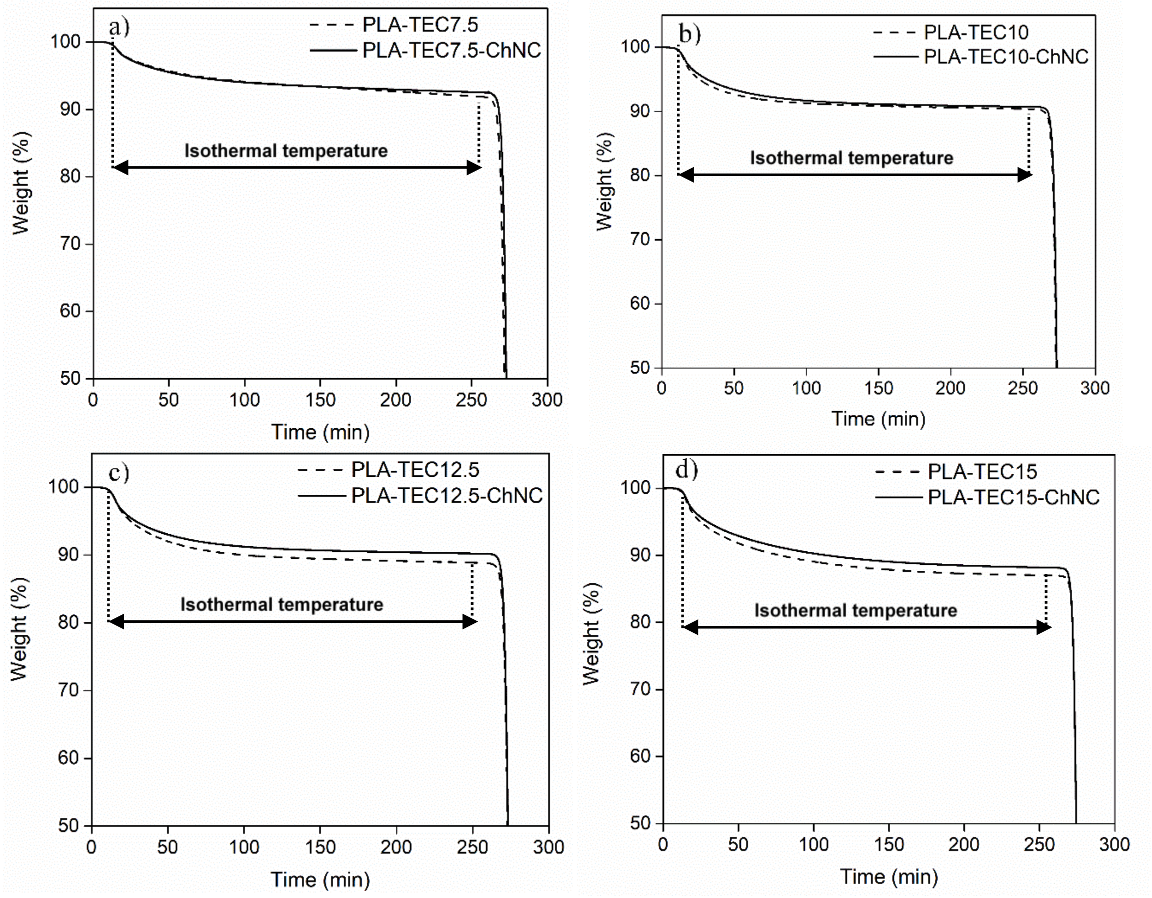

Figure S1. Isothermal TGA graphs of PLA-TEC and its nanocomposite with different TEC contents. a) 7.5%, b) 10%, c) 12.5%, d) 15%.

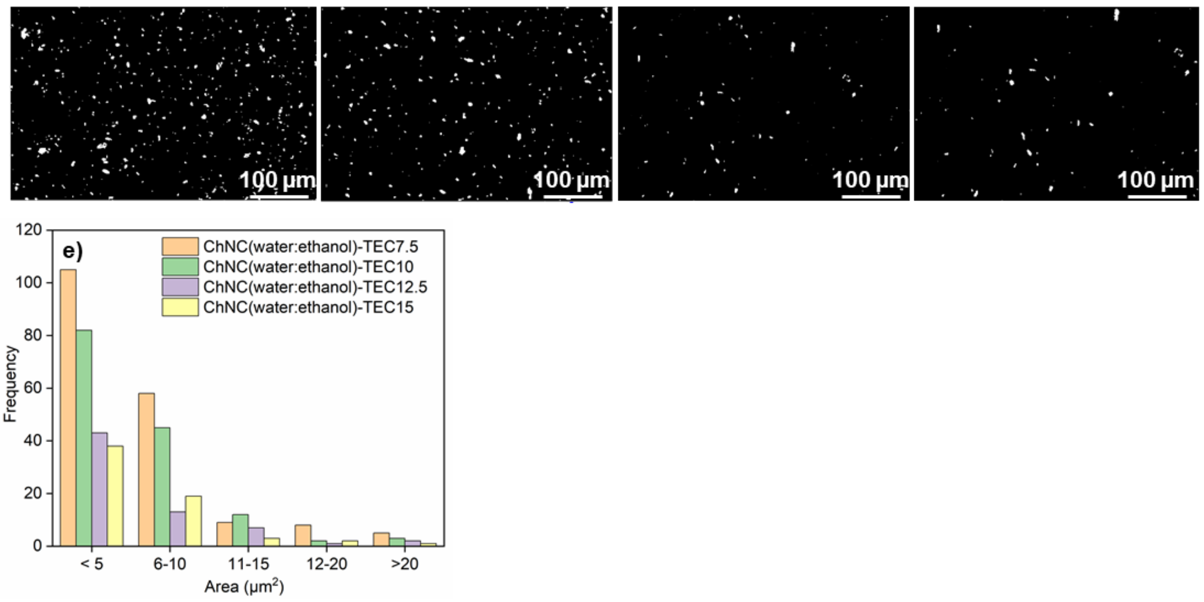

Figure S2. Image analysis using ImageJ software of the ChNC dispersion in the feeding suspensions with varied TEC contents a) 7.5% b) 10%, c) 12.5%, d) 15% e) size distribution of the ChNC-aggregates.

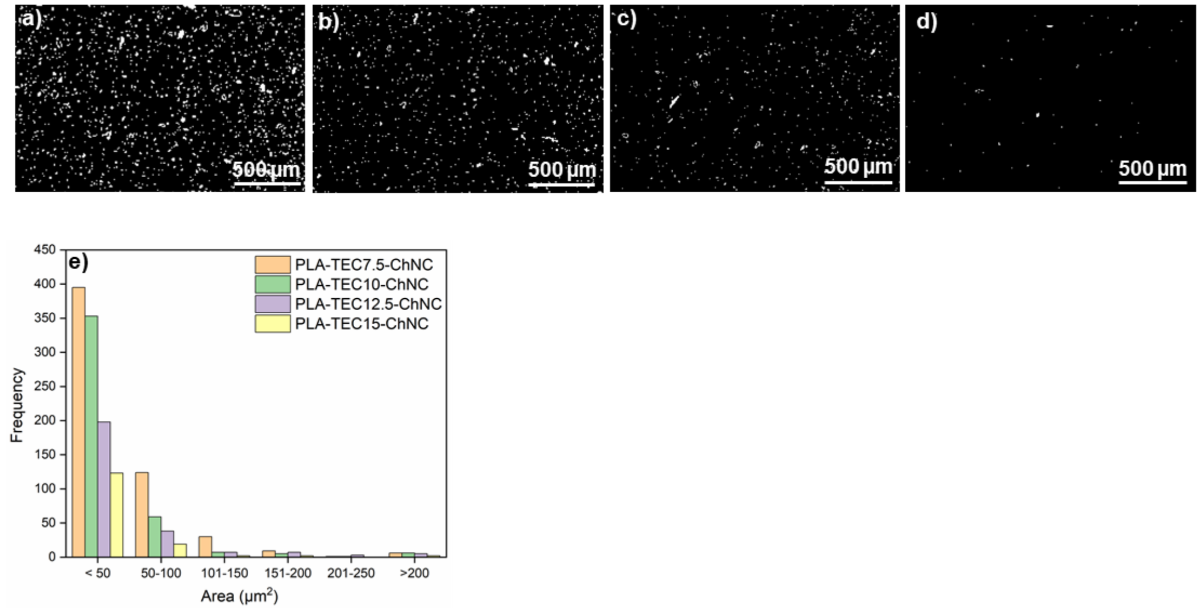

Figure S3. Image analysis using ImageJ software of the ChNC dispersions in the nanocomposites with varied TEC contents. a) 7.5%, b) 10%, c) 12.5%, d) 15%, e) size distribution of the ChNC-aggregates.

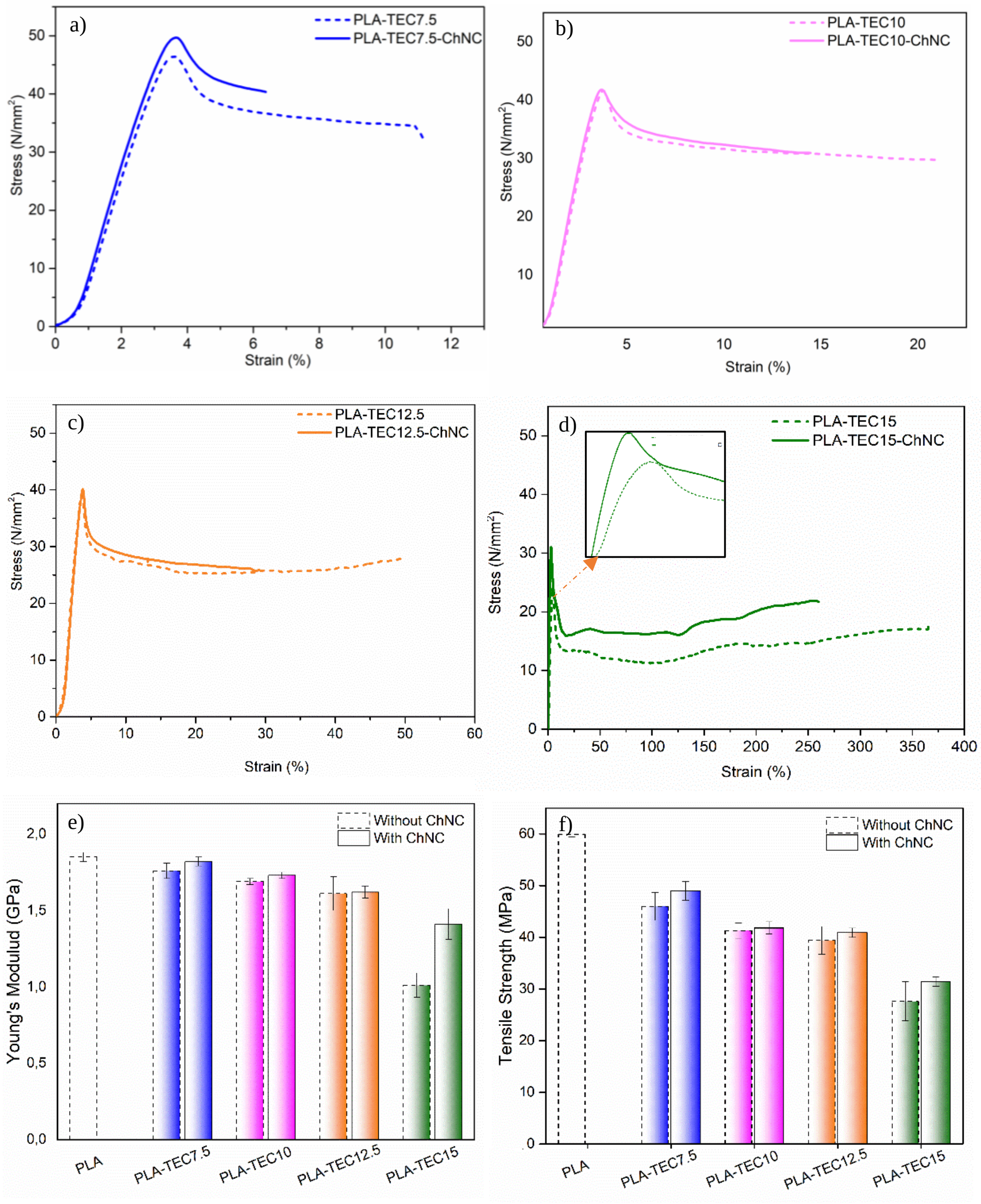

Figure S4. Typical stress-strain graphs of PLA-TEC with and without ChNCs. a) TEC7.5%, b) TEC10%, c) TEC 12.5%TEC, d) TEC15%, e) Young's modulus and f) tensile strength.
